# A comparison of the National Surgical Quality Improvement Program and the Society of Thoracic Surgery Cardiac Surgery preoperative risk models: a cohort study

**DOI:** 10.1097/JS9.0000000000000490

**Published:** 2023-05-18

**Authors:** Adam R. Dyas, Michael R. Bronsert, William G. Henderson, Christina M. Stuart, Nisha Pradhan, Kathryn L. Colborn, Joseph C. Cleveland, Robert A. Meguid

**Affiliations:** a Department of Surgery; b Surgical Outcomes and Applied Research Program; c Adult and Child Center for Health Outcomes Research and Delivery Science; d Department of Medicine, University of Colorado School of Medicine; e Department of Biostatistics and Informatics, Colorado School of Public Health, Aurora, CO, USA

**Keywords:** ACS-NSQIP, cardiac surgery, NSQIP, postoperative outcomes, STS-ACSD, ACSD

## Abstract

**Background:**

Cardiac surgery prediction models and outcomes from the American College of Surgeons National Surgical Quality Improvement Program (ACS-NSQIP) have not been reported. The authors sought to develop preoperative prediction models and estimates of postoperative outcomes for cardiac surgery using the ACS-NSQIP and compare these to the Society of Thoracic Surgeons Adult Cardiac Surgery Database (STS-ACSD).

**Methods:**

In a retrospective analysis of the ACS-NSQIP data (2007–2018), cardiac operations were identified using cardiac surgeon primary specialty and sorted into cohorts of coronary artery bypass grafting (CABG) only, valve surgery only, and valve+CABG operations using CPT codes. Prediction models were created using backward selection of the 28 non-laboratory preoperative variables in ACS-NSQIP. Rates of nine postoperative outcomes and performance statistics of these models were compared to published STS 2018 data.

**Results:**

Of 28 912 cardiac surgery patients, 18 139 (62.8%) were CABG only, 7872 (27.2%) were valve only, and 2901 (10.0%) were valve+CABG. Most outcome rates were similar between the ACS-NSQIP and STS-ACSD, except for lower rates of prolonged ventilation and composite morbidity and higher reoperation rates in ACS-NSQIP (all *P*<0.0001). For all 27 comparisons (9 outcomes × 3 operation groups), the c-indices for the ACS-NSQIP models were lower by an average of ~0.05 than the reported STS models.

**Conclusions:**

The ACS-NSQIP preoperative risk models for cardiac surgery were almost as accurate as the STS-ACSD models. Slight differences in c-indexes could be due to more predictor variables in STS-ACSD models or the use of more disease- and operation-specific risk variables in the STS-ACSD models.

## Graphical abstract

**Figure:**
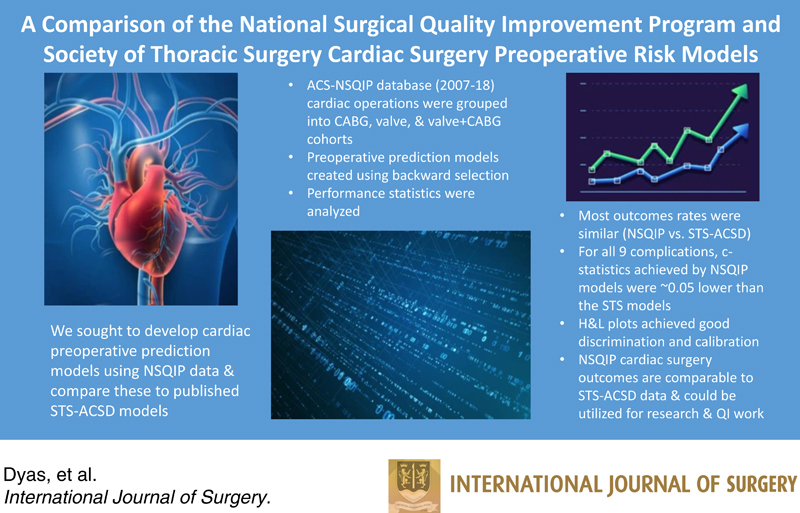

## Introduction

Highlights

- We used the American College of Surgeons National Surgical Quality Improvement Program (ACS-NSQIP) to develop cardiac surgery preoperative risk models.
- The models generated achieved almost the save predictive capabilities as the Society of Thoracic Surgeons Adult Cardiac Surgery Database (STS-ACSD).
- These models would be beneficial for institutions participating in the ACS-NSQIP who would like to perform quality improvement for cardiac surgery.
- Data Statement
- The analysis from this study used deidentified and publicly available data from the American College of Surgeons Participant Use File. Therefore, the dataset will not be provided with the manuscript submission.

Programs for comparing risk-adjusted surgical outcomes are important for quality improvement (QI) efforts. Two early surgical QI programs using risk-adjusted outcomes were the Veterans Affairs Continuous Improvement in Cardiac Surgery Program^1,2^ and the Society of Thoracic Surgeons Adult Cardiac Surgery Database (STS-ACSD)^3^. The STS-ACSD includes more than 7.5 million cardiac operations and has served as the gold standard for risk-adjusted postoperative outcomes for cardiac surgery since the original development of its risk prediction models^4–8^. The STS short-term postoperative risk calculator requires manual input of more than 50 variables to predict patient outcomes, and only uses data in their cardiac models from institutions within the United States and Canada. Predictor variables include patient demographics, medical comorbidities, recent procedures, and laboratory values^9,10^. The STS-ACSD mainly uses disease- and operation-specific preoperative variables to predict postoperative outcomes and calculate risk-adjusted outcomes. Institutions benefit from the STS-ACSD because it allows them to evaluate their specific risk-adjusted rates of postoperative outcomes compared to other participating institutions. If an institution finds that it is a low performer in a certain postoperative complication (e.g. mediastinitis/deep sternal wound infection), then more resources can be allocated and efforts be focused toward improving this outcome (e.g. use of negative pressure wound vacs, changes to perioperative antibiotic prophylaxis, etc.).

Based on the success of cardiac surgery QI programs, similar programs were developed for other surgical specialties. The Department of Veterans Affairs started the National Surgical Quality Improvement Program (VA-NSQIP)^11^, which was subsequently adopted for civilian operations by the American College of Surgeons (ACS)^12^. The ACS-NSQIP originally encompassed nine non-cardiac surgical specialties. Therefore, by necessity, it collected mainly generic preoperative variables and postoperative outcomes that would be applicable in a broad surgical population for the prediction of postoperative outcomes and risk-adjustment. In 2007, the ACS-NSQIP also started collecting data on cardiac operations. This provides the opportunity to compare outcomes of cardiac surgery between the STS-ACSD and ACS-NSQIP, and also to compare risk models using mainly disease-specific and operation-specific predictor variables vs. more generic predictor variables. While the ACS-NSQIP has long been considered the gold standard for surgical reporting and QI efforts in non-cardiac operations^13^, the cardiac surgery data within the database have never been comprehensively analyzed. We found only one study in the literature that analyzed the postoperative outcomes of cardiac surgery in the ACS-NSQIP and compared them to outcomes in the STS-ACSD^14^. However, this was a single institution study and did not attempt to generate preoperative prediction models. If models using more generic variables in a more widely available database were found to be similarly predictive of postoperative outcomes, more institutions would have access to these data and have the ability to perform quality improvement, especially internationally. For example, an institution participating in the cardiac ACS-NSQIP could identify their cases in the ACS-NSQIP database, and compare their risk-adjusted outcomes to those of the other institutions participating in the cardiac ACS-NSQIP.

The purpose of this study was to estimate outcome rates and develop preoperative prediction models for postoperative cardiac surgery outcomes in the ACS-NSQIP, comparing these to the gold standard STS-ACSD. We hypothesized that cardiac surgical outcomes in the ACS-NSQIP would be similar to those in the STS-ACSD, and that the preoperative prediction models created using the generic variables of the ACS-NSQIP participant use file (PUF) would achieve similar performance characteristics as those generated using the disease-specific and operation-specific variables of the STS-ACSD. This would be important for the more than 40 institutions outside of North America who participate in NSQIP because they could use these data to perform quality improvement efforts in cardiac surgery.

## Patients and methods

### Study design and patients

This was a retrospective analysis of the prospectively collected ACS-NSQIP cardiac data, 2007–2018. This work has been reported in line with the Strengthening the Reporting of Cohort Studies in Surgery (STROCSS) criteria^15^, Supplemental Digital Content 1, http://links.lww.com/JS9/A510. Because the ACS-NSQIP data are deidentified and publicly available, the study was deemed exempt from review by the Colorado Multiple Institutional Review Board.

The ACS-NSQIP data are collected from over 700 hospitals throughout the US and internationally, including academic referral centres, private-based hospitals, and hospitals in both urban and rural settings. Data are collected by trained ACS clinical nurses and are audited. The STS-ACSD uses these categories of cardiac surgery in their statistical analyses and reporting: coronary artery bypass grafting (CABG) only; valve surgery only [including aortic valve replacement (AVR) only, mitral valve replacement (MVR) only, mitral valve repair (MVr) only, MVR/MVr, and AVR and MVR/MVr]; and valve plus CABG (valve+CABG) surgery (including AVR and CABG, MVR and CABG, and MVr and CABG); and other cardiac operations^10^. We identified the CPT codes for each of these groups and generated similar patient cohorts using the CPT codes in the ACS-NSQIP. We began by including all patients who underwent operations performed by surgeons whose specialty was designated as cardiac surgery. Patients who had incomplete data, who did not have operations with CPT codes for surgical procedures on the heart and pericardium (CPT codes 33016–33999), or who had cardiac operations other than CABG only, valve repair or replacement only, or valve+CABG were excluded. We classified the cardiac surgery cases into the different operation types as follows: CABG only (CPT codes 33508, 33510–33536, 35500, 35572, 35600); AVR only (33405–33412); MVR only (33430); and MVr (33418–33427).

The ACS-NSQIP collects 28 non-laboratory preoperative predictor variables that are appropriate for a broad surgical population (e.g. demographic variables; general comorbidities such as diabetes, hypertension, history of congestive heart failure, etc.; functional health status; American Society of Anesthesiology physical status classification (ASA class); emergency operation; inpatient/outpatient setting, etc.). The ACS-NSQIP preoperative laboratory variables were not used because we previously found that they did not add significant prediction beyond the non-laboratory preoperative variables, and they were often missing not at random^16^.

We used the ACS-NSQIP to generate rates of the following nine postoperative outcomes reported by the STS-ACSD: in-hospital mortality (which also includes any 30-day mortality for patients discharged prior to 30 days); stroke; renal complication; prolonged ventilation (>48 h); unplanned reoperation; composite morbidity and mortality (defined as the occurrence of any of the previous complications); prolonged postoperative length of stay (PLOS >14 days); short PLOS (PLOS < 6 days and patient alive at discharge); and mediastinitis/deep sternal wound infection (DSWI)^10^. The STS-ACSD and ACS-NSQIP track all outcomes up to 30 days postoperatively.

### Statistical analysis

The ACS-NSQIP patient non-laboratory preoperative characteristics were compared between the CABG only, valve only, and valve+CABG groups by χ^2^ test for the categorical variables and analysis of variance for the continuous variables. Unadjusted rates of the nine postoperative outcomes were compared between the STS-ACSD and the ACS-NSQIP PUF using χ^2^ tests. Risk-adjusted rates could not be compared between the two databases because we did not have access to the STS-ACSD data. To create preoperative risk models for the nine postoperative outcomes from the ACS-NSQIP, we used backward stepwise logistic regression analysis with each postoperative outcome (yes/no) as the independent variable and the preoperative non-laboratory ACS-NSQIP variables as the dependent variables with an exit criterion of *P* value greater than 0.05. The resulting parsimonious models were then compared in a one-step method to approximate estimation of leave one out cross validation to assess the generalizability of the resulting models. We used the resulting predicted probabilities and cross validation predicted estimates to calculate the area under the receiver operating characteristic curves and 95% CI using the Delong method. We calculated Brier scores and constructed Hosmer–Lemeshow (H&L) graphs of observed to expected values for evaluation of calibration. The rates of the nine outcomes and the c-indexes of the models for the STS-ACSD were from the 2018 STS-ACSD publication^10^. Two-sided *P* values less than or equal to 0.05 were considered statistically significant. All statistical analyses were performed using SAS version 9.4 (SAS Inc).

## Results

There were 38 650 patients in the ACS-NSQIP PUF from 2007 to 2018 who had operations by cardiac surgeons. Of these, 4151 patients (10.8%) were excluded for having operations outside of the list of cardiac CPT codes (33016–33999), 706 (1.8%) were excluded for missing data, and 4881 (12.6%) were excluded for having cardiac operations other than CABG only, valve only, or valve+CABG. Of the resulting 28 912 patients (74.8%) included in the analysis cohort, 18 139 (62.8%) were CABG only, 7872 (27.2%) were valve only, and 2901 (10.0%) were valve+CABG. This distribution was close to the distribution reported by the STS in 2018: 65.9% CABG only; 22.4% valve only; and 11.7% valve+CABG^10^.

Table 1 shows the preoperative characteristics of the included patient cohort. Patients undergoing cardiac surgery were mostly white, older than 60 years of age, had one or more medical comorbidities, and were ASA class III or IV. Patients undergoing CABG only, compared to valve only or valve+CABG, tended to have a higher percentage of males, a history of diabetes, smoking, and bleeding disorders, and were more likely to be transferred from an acute care hospital. Patients undergoing valve only tended to have a higher percentage of females, with dyspnoea, hypertension, and having a more complex operation at a higher work RVU. Patients undergoing valve+CABG tended to be older, and more often with chronic obstructive pulmonary disease, congestive heart failure, and classified as ASA class IV. All patient characteristics had statistically significant differences between the three operation groups, except for the preoperative comorbidities of acute renal failure, on dialysis, and having disseminated cancer, which were relatively equal across the operation groups.

**Table 1:** Patient demographics and comorbidities of the ACS-NSQIP cardiac surgery patient cohort for the three STS-ASCD categories (*n*=28 912).

	Total	CABG only	Valve only	Valve+CABG	
	*n*=28 912	*n*=18 139	*n*=7872	*n*=2901	
Patient characteristics	*n*, column %	*n*, column %	*n*, column %	*n*, column %	*P*
Sex					<0.0001
Male	20 577 (71.2)	13 900 (76.6)	4611 (58.6)	2066 (71.2)	
Female	8335 (28.8)	4239 (23.4)	3261 (41.4)	835 (28.8)	
Age, mean (SD)	66.2 (11.1)	65.7 (10.2)	65.5 (13.5)	71.5 (9.9)	<0.0001
Race/ethnicity					<0.0001
American or Alaskan Native	131 (0.5)	95 (0.5)	30 (0.4)	6 (0.2)	
Asian or Pacific Islander	850 (2.9)	648 (3.6)	160 (2.0)	42 (1.4)	
Black	1428 (4.9)	922 (5.1)	405 (5.1)	101 (3.5)	
Hispanic	2068 (7.2)	1418 (7.8)	520 (6.6)	130 (4.5)	
White	17 352 (60.0)	10 455 (57.6)	5091 (64.7)	1806 (62.3)	
Unknown	7083 (24.5)	4601 (25.4)	1666 (21.2)	816 (28.1)	
Body mass index (kg/m^2^)					<0.0001
<18.5	217 (0.8)	94 (0.5)	104 (1.3)	19 (0.7)	
18.5–24.9	5909 (20.4)	3244 (17.9)	2029 (25.8)	636 (21.9)	
25–29.9	10 622 (36.7)	6768 (37.3)	2769 (35.2)	1085 (37.4)	
30–34.9	7236 (25.0)	4857 (26.8)	1679 (21.3)	700 (24.1)	
35–39.9	2982 (10.3)	1963 (10.8)	723 (9.2)	296 (10.2)	
>40	1711 (5.9)	1042 (5.7)	520 (6.6)	149 (5.1)	
Unknown	235 (0.8)	171 (0.9)	48 (0.6)	16 (0.6)	
Diabetes mellitus					<0.0001
Insulin	4016 (13.9)	3107 (17.1)	531 (6.7)	378 (13.0)	
Oral medications	5648 (19.5)	4145 (22.9)	937 (11.9)	566 (19.5)	
None	19 248 (66.6)	10 877 (60.0)	6404 (81.4)	1957 (67.5)	
Dyspnoea					<0.0001
At rest	1504 (5.2)	812 (4.5)	516 (6.6)	176 (6.1)	
With moderate exertion	10 143 (35.1)	4989 (27.5)	3850 (48.9)	1304 (45.0)	
None	17 265 (59.7)	12 338 (68.0)	3506 (44.5)	1421 (49.0)	
Functional health status					0.0006
Independent	27 660 (95.7)	17 301 (95.4)	7554 (96.0)	2805 (96.7)	
Partially dependent	978 (3.4)	670 (3.7)	231 (2.9)	77 (2.7)	
Totally dependent	274 (1.0)	168 (0.9)	87 (1.1)	19 (0.7)	
Smoker within 1 year					<0.0001
Yes	5527 (19.1)	4020 (22.2)	1066 (13.5)	441 (15.2)	
No	23 385 (80.9)	14 119 (77.8)	6806 (86.5)	2460 (84.8)	
Ventilator dependent					<0.0001
Yes	328 (1.1)	165 (0.9)	119 (1.5)	44 (1.5)	
No	28 584 (98.9)	17 974 (99.1)	7753 (98.5)	2857 (98.5)	
History of COPD					0.0004
Yes	2440 (8.4)	1471 (8.1)	670 (8.5)	299 (10.3)	
No	26 472 (91.6)	16 668 (91.9)	7202 (91.5)	2602 (89.7)	
Hypertension requiring medication					<0.0001
Yes	6351 (22.0)	3214 (17.7)	2593 (32.9)	544 (18.8)	
No	22 561 (78.0)	14 925 (82.3)	5279 (67.1)	2357 (81.2)	
History of congestive heart failure					<0.0001
Yes	4053 (14.0)	1814 (10.0)	1550 (19.7)	689 (23.8)	
No	24 859 (86.0)	16 325 (90.0)	6322 (80.3)	2212 (76.2)	
Presence of ascites					<0.0001
Yes	37 (0.1)	9 (<0.1)	21 (0.3)	7 (0.2)	
No	28 875 (99.9)	18 130 (99.9)	7851 (99.7)	2894 (99.8)	
Acute renal failure					0.32
Yes	204 (0.7)	137 (0.8)	46 (0.6)	21 (0.7)	
No	28 708 (99.3)	18 002 (99.2)	7826 (99.4)	2880 (99.3)	
Dialysis					0.73
Yes	744 (2.6)	461 (2.5)	202 (2.6)	81 (2.8)	
No	28 168 (97.4)	17 678 (97.5)	7670 (97.4)	2820 (97.2)	
Presence of disseminated cancer					0.56
Yes	60 (0.2)	34 (0.2)	20 (0.3)	6 (0.2)	
No	28 852 (99.8)	18 105 (99.8)	7852 (99.7)	2895 (99.8)	
Open wound or wound infection					0.01
Yes	409 (1.4)	263 (1.4)	91 (1.6)	55 (1.9)	
No	28 503 (98.6)	17 876 (98.6)	7781 (98.8)	2846 (98.1)	
Chronic steroid use					0.0002
Yes	810 (2.8)	464 (2.6)	234 (3.0)	112 (3.9)	
No	28 102 (97.2)	17 675 (97.4)	7638 (97.0)	2789 (96.1)	
More than 10% weight loss within 6 months					<0.0001
Yes	199 (0.7)	72 (0.4)	102 (1.3)	25 (0.9)	
No	28 713 (99.3)	18 067 (99.6)	7770 (98.7)	2876 (99.1)	
Bleeding disorder					<0.0001
Yes	2987 (10.3)	2230 (12.3)	493 (6.3)	264 (9.1)	
No	25 925 (89.7)	15 909 (87.7)	7379 (93.7)	2637 (90.9)	
Blood transfusion within 72 h					<0.0001
Yes	536 (1.9)	318 (1.8)	134 (1.7)	84 (2.9)	
No	28 376 (98.2)	17 821 (98.2)	7738 (98.3)	2817 (97.1)	
Presence of systemic sepsis					<0.0001
Septic shock	82 (0.3)	10 (0.1)	65 (0.8)	7 (0.2)	
Sepsis	132 (0.5)	28 (0.2)	91 (1.2)	13 (0.4)	
SIRS	740 (2.6)	488 (2.7)	174 (2.2)	78 (2.7)	
None	27 958 (96.7)	17 613 (97.1)	7542 (95.8)	2803 (96.6)	
Transfer status					<0.0001
Acute care hospital	5293 (18.3)	4069 (22.4)	722 (9.2)	502 (17.3)	
Chronic care facility	101 (0.4)	52 (0.3)	36 (0.5)	13 (0.4)	
Admitted from home	23 518 (81.3)	14 018 (77.3)	7114 (90.4)	2386 (82.2)	
ASA class					<0.0001
I—no disturbance	33 (0.1)	14 (0.1)	15 (0.2)	4 (0.1)	
II—mild disturbance	103 (0.4)	54 (0.3)	43 (0.5)	6 (0.2)	
III—severe disturbance	5539 (19.2)	3313 (18.3)	1812 (23.0)	414 (14.3)	
IV—life threatening disturbance	23 029 (79.7)	14 636 (80.7)	5942 (75.5)	2451 (84.5)	
V—moribund patient	208 (0.7)	122 (0.7)	60 (0.8)	26 (0.9)	
Work RVU, mean (SD)	35.4 (10.0)	31.6 (11.8)	45.8 (5.8)	42.9 (10.0)	<0.0001

ACS-NSQIP, American College of Surgeons National Surgical Quality Improvement Program; ASA class, American Society of Anesthesiology physical status classification; CABG, coronary artery bypass grafting; COPD, chronic obstructive pulmonary disease; RVU, relative value unit; SIRS, systemic inflammatory response system; STS-ACSD, Society of Thoracic Surgeons Adult Cardiac Surgery Database.

Table 2 shows the comparison of unadjusted postoperative outcome rates between the ACS-NSQIP and the STS-ACSD. Most outcome rates, even when statistically different, were clinically similar between the two databases, except for lower rates in the ACS-NSQIP for prolonged ventilation (CABG only: 5.04% ACS-NSQIP vs. 9.33% STS-ACSD; valve only: 6.61% vs. 11.06%; valve+CABG: 11.62% vs. 18.88%, all *P*<0.0001) and composite morbidity and mortality (CABG only: 11.94 vs. 14.98%; valve only: 15.84% vs. 18.37%; valve+CABG: 22.99% vs. 28.30%, all *P*<0.0001) and higher rates of reoperation (CABG only: 5.39% vs. 2.35%; valve only: 8.07% vs. 4.24%; valve+CABG: 10.44% vs. 5.12%, all *P*<0.0001) compared with the STS-ACSD.

**Table 2:** Comparison of patient postoperative outcomes rates observed in the ACS-NSQIP PUF vs. the STS-ACSD.

	CABG only			Valve only			Valve+CABG		
Patient Outcomes	STS *n*=439 092	ACS-NSQIP *n*=18 139	*P* value	STS *n*=150 150	ACS-NSQIP *n*=7872	*P* value	STS n=81 588	ACS-NSQIP *n*=2901	*P* value
Mortality	2.37%	2.35%	0.86	3.17%	2.79%	0.06	5.61%	4.52%	0.01
	8852/ 373 683	426/ 18 139		4004/ 126 204	220/ 7872		3936/ 70 111	131/ 2901	
Stroke	1.28%	1.67%	<0.0001	1.49%	1.68%	0.19	2.47%	3.00%	0.07
	5621/ 438 385	303/ 18 139		2237/ 149 800	132/ 7872		2008/ 81 376	87/ 2901	
Renal failure	2.21%	1.66%	<0.0001	2.66%	2.32%	0.07	5.04%	3.83%	0.003
	9381/ 424 888	301/ 18 139		3868/ 145 454	183/ 7872		3953/ 78 466	111/ 2901	
Prolonged ventilation	9.33%	5.04%	<0.0001	11.06%	6.61%	<0.0001	18.88%	11.62%	<0.0001
	40 974/ 439 092	915/ 18 139		16 604/ 150 150	520/ 7872		15 406/ 81 588	337/ 2901	
Reoperation	2.35%	5.39%	<0.0001	4.24%	8.07%	<0.0001	5.12%	10.44%	<0.0001
	10 327/ 439 060	977/ 18 139		6371/ 150 137	635/ 7872		4174/ 81 581	303/ 2901	
Morbidity and mortality	14.98%	11.94%	<0.0001	18.37%	15.84%	<0.0001	28.30%	22.99%	<0.0001
	56 984/ 380 491	2165/ 18 139		23 724/ 129 140	1247/ 7872		20 472/ 72 345	667/ 2901	
Prolonged PLOS	5.03%	6.20%	<0.0001	7.96%	9.46%	<0.0001	12.88%	15.10%	0.0005
	22 091/ 438 867	1125/ 18 139		11 941/ 150 024	745/ 7872		10 501/ 81 537	438/ 2901	
Short PLOS	48.27%	41.60%	<0.0001	37.41%	33.82%	<0.0001	22.58%	21.30%	0.11
	211 820/ 438 867	7545/ 18 139		56 130/ 150 024	2662/7872		18 412/ 81 537	618/ 2901	
DSWI	0.31%	0.88%	<0.0001	0.16%	0.53%	<0.0001	0.35%	0.79%	0.0001
	1346/ 438 270	160/ 18 139		244/ 149 778	42/ 7872		285/ 81 344	23/ 2901	

ACS-NSQIP PUF, American College of Surgeons National Surgical Quality Improvement Program Participant Use File; CABG, coronary artery bypass grafting; DSWI, deep sternal wound infection; PLOS, postoperative length of stay; STS-ASCD, Society of Thoracic Surgeons Adult Surgery Cardiac Database.

Table 3 shows the performance statistics of 27 models (nine outcomes for each of the three operation groups) in the training and testing datasets using the ACS-NSQIP. In general, the best models (highest c-index and lowest Brier score) were in the CABG cohort (average of c-indexes in test set=0.684, average Brier score=0.060), followed by the valve cohort (0.678, 0.068), followed by valve+CABG (0.654, 0.083).

**Table 3:** Performance of the 27 models using the ACS-NSQIP PUF in the training and testing datasets (*n*=28 912).

	CABG only				Valve only				CABG + valve			
	Training set		Testing set		Training set		Testing set		Training set		Testing set	
Patient endpoints	c-index	Brier score	c-index	Brier score	c-index	Brier score	c-index	Brier score	c-index	Brier score	c-index	Brier score
Mortality	0.749	0.022	0.739	0.022	0.729	0.026	0.713	0.027	0.722	0.041	0.695	0.042
Stroke	0.663	0.016	0.648	0.016	0.682	0.016	0.655	0.016	0.593	0.029	0.571	0.029
Renal failure	0.744	0.016	0.731	0.016	0.765	0.022	0.753	0.022	0.732	0.035	0.711	0.036
Prolonged ventilation	0.737	0.045	0.729	0.045	0.749	0.056	0.742	0.056	0.735	0.091	0.714	0.094
Reoperation	0.625	0.050	0.616	0.050	0.605	0.073	0.592	0.074	0.582	0.093	0.567	0.093
Morbidity and mortality	0.683	0.098	0.678	0.098	0.683	0.122	0.677	0.123	0.661	0.163	0.650	0.165
Prolonged PLOS	0.707	0.055	0.701	0.056	0.737	0.078	0.728	0.079	0.691	0.119	0.677	0.121
Short PLOS	0.665	0.224	0.662	0.225	0.675	0.206	0.670	0.207	0.649	0.160	0.637	0.162
DSWI	0.682	0.009	0.652	0.009	0.709	0.005	0.572	0.005	0.775	0.008	0.664	0.008
Overall mean	0.695	0.059	0.684	0.060	0.704	0.067	0.678	0.068	0.682	0.082	0.654	0.083

ACS-NSQIP PUF, American College of Surgeons National Surgical Quality Improvement Program Participant Use File; CABG, coronary artery bypass grafting; DSWI, deep sternal wound infection; PLOS, postoperative length of stay.

Table 4 compares the testing dataset c-indexes of the 27 preoperative prediction models calculated from the ACS-NSQIP and STS-ACSD databases. All of the ACS-NSQIP models had lower c-indexes than the STS-ACSD models, except for DSWI in the valve+CABG model. The difference in mean c-index in STS-ACSD and ACS-NSQIP models for the CABG cohort was 0.053, for the valve cohort was 0.047, and for the valve+CABG cohort was 0.048, indicating slightly lower discrimination of the ACS-NSQIP models compared to the STS-ACSD models. The STS-ACSD c-index was within the 95% CI of the c-index for the ACS-NSQIP for two of nine CABG models (reoperation and DSWI), two of nine valve models (stroke and renal failure), and five of nine valve+CABG models (stroke, renal failure, prolonged ventilation, reoperation, DSWI).

**Table 4:** Comparison of preoperative risk model testing set c-indexes between ACS-NSQIP PUF and STS-ACSD.

	CABG only		Valve only		Valve+CABG	
Patient Outcomes	STS *n*=439 092	ACS-NSQIP *n*=18 139 c-index (95% CI)	STS *n*=150 150	ACS-NSQIP *n*=7872 c-index (95% CI)	STS *n*=81 588	ACS-NSQIP *n*=2901 c-index (95% CI)
Mortality	0.804	0.739 (0.714–0.763)	0.775	0.713 (0.676–0.749)	0.761	0.695 (0.648–0.741)
Stroke	0.697	0.648 (0.618–0.678)	0.656	0.655 (0.607–0.704)	0.632	0.571 (0.510–0.633)
Renal failure	0.826	0.731 (0.699–0.762)	0.787	0.753 (0.714–0.792)	0.759	0.711 (0.662–0.759)
Prolonged ventilation	0.772	0.729 (0.712–0.747)	0.777	0.742 (0.719–0.764)	0.744	0.714 (0.685–0.744)
Reoperation	0.621	0.616 (0.598–0.635)	0.616	0.592 (0.569–0.615)	0.588	0.567 (0.533–0.602)
Morbidity and mortality	0.738	0.678 (0.665–0.690)	0.723	0.677 (0.660–0.694)	0.712	0.650 (0.625–0.674)
Prolonged PLOS	0.777	0.701 (0.685–0.717)	0.796	0.728 (0.709–0.748)	0.739	0.677 (0.650–0.704)
Short PLOS	0.716	0.662 (0.654–0.670)	0.732	0.670 (0.657–0.682)	0.726	0.637 (0.613–0.662)
DSWI	0.681	0.652 (0.607–0.696)	0.665	0.572 (0.594–0.655)	0.659	0.664 (0.547–0.780)
Overall (mean)	0.737	0.684	0.725	0.678	0.702	0.654

ACS-NSQIP PUF, American College of Surgeons National Surgical Quality Improvement Program Participant Use File; CABG, coronary artery bypass grafting; DSWI, deep sternal wound infection; PLOS, postoperative length of stay; STS-ASCD, Society of Thoracic Surgeons Adult Surgery Cardiac Database.

**Figure 1:**
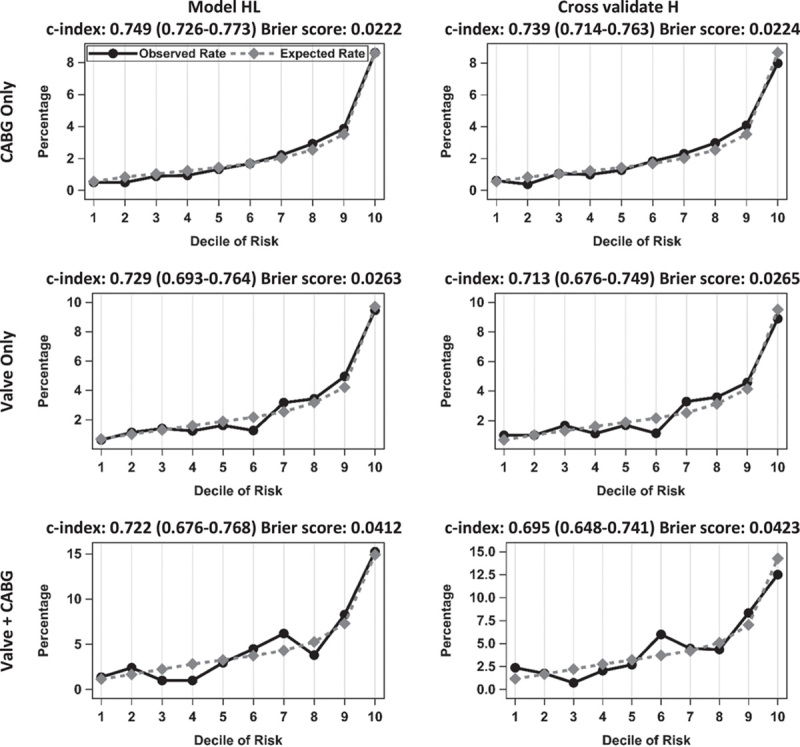
Hosmer–Lemeshow plots of expected to observed rates of postoperative mortality within the three patient cohorts. CABG, coronary artery bypass graft; HL, Hosmer–Lemeshow.

Figure 1 shows the H&L calibration plots computed from the ACS-NSQIP data for mortality for each of the three cohorts, and supplemental Figure 1-3, Supplemental Digital Content 2, http://links.lww.com/JS9/A511, Supplemental Digital Content 3, http://links.lww.com/JS9/A512, Supplemental Digital Content 4, http://links.lww.com/JS9/A513 show the H&L plots for the remainder of the postoperative outcomes of the CABG only, valve only, and valve+CABG cohorts, respectively. The H&L plots showed good calibration for the outcomes in the CABG only and valve only cohorts. Calibration was not as good in the valve+CABG cohort likely because of the cohort’s small sample size.

## Discussion

We successfully used the ACS-NSQIP cardiac surgery data to develop preoperative prediction models and estimate unadjusted incidence rates for the nine postoperative outcomes within the three cardiac surgery patient cohorts used by the STS-ACSD. We believe that this is the first comprehensive analysis of the cardiac surgery data in the ACS-NSQIP and the first comprehensive comparison of these data to the data in the STS-ACSD. While the patient populations outside of North America are likely different in demographic factors, medical comorbidities, and frequency of cardiac operations performed, these models could be beneficial for institutions outside of North America who participate in the ACS-NSQIP but not the STS-ACSD hoping to perform quality improvement activities in cardiac surgery.

We found that the distributions of CABG only, valve only, and valve+CABG operations were similar between the ACS-NSQIP and STS-ACSD databases. The unadjusted rates of the nine postoperative outcomes were clinically similar between the two databases, except for lower rates of prolonged ventilation and composite mortality/major morbidity, and higher rates of reoperation, in the ACS-NSQIP. The prolonged ventilation differences were likely due to differences in definition of “prolonged,” which the ACS-NSQIP defines as more than 48 h while the STS-ACSD defines as more than 24 h. The higher rates of reoperation in the ACS-NSQIP were likely because ACS-NSQIP counts any reoperation in the 30 days after the patient’s cardiac operation while the STS-ACSD only considers cardiac reoperations. We were unable to compare risk-adjusted outcome rates between the two databases, because we did not have access to the STS-ACSD data. Finally, we found that the ACS-NSQIP preoperative prediction models performed almost as well as the STS-ACSD models but were slightly less predictive. This was probably due to the STS-ACSD models having larger sample sizes, more predictor variables, and predictor variables that were disease-specific rather than generic. The H&L plots for the ACS-NSQIP models reflected good calibration for the CABG only and valve only cohorts but weaker calibration in the valve+CABG cohort. This study shows that generic surgical variables can be effectively used to create preoperative predictions models for complications in cardiac surgery. Both of these models are freely available and can be used globally to calculate operative risk of patients undergoing these operations (https://riskcalculator.facs.org; https://riskcalc.sts.org>stswebriskcalc).

The STS-ASCD remains the gold standard cardiac surgical outcomes database. Since implementation in 1989, regular audit shows greater than 95% concordance of the data inputted to the STS with source data^3^. It has been used to develop the online STS Short-Term Risk Calculator for estimating risk of postoperative complications after common cardiac operations^9,10,17^, which aids in informed decision making. There have been other attempts to develop preoperative predictor models for cardiac surgery patients besides the STS-ASCD. A review paper by Prins *et al*.^18^. details 19 historical cardiac surgery models including the EuroSCORE^19,20^ and the Parsonnet score^21^. The EuroSCORE was developed from only European patients and only predicts postoperative mortality, which may not be generalizable to other patients and is less useful than the ability to predict other outcomes. The Parsonnet score has fallen out of favour since the early 2000s due to diminished predictive accuracy^22^.

The information obtained in this study could be useful for institutions who participate in ACS-NSQIP and do not have access to the STS-ACSD. While the STS-ACSD has achieved ~95% institutional penetrance in the United States among Centers for Medicare and Medicaid Services institutions, this leaves more than 50 institutions without access to the STS-ACSD in the USA alone^23^. Department of Defense (DoD) hospitals, which participate in the ACS-NSQIP, might also benefit from non-STS cardiac surgery data. Additionally, the ACS-NSQIP has at least 40 participating institutions in Europe and Asia. Since less than ten institutions outside of North America participate in the STS-ACSD, these models using the ACS-NSQIP cardiac surgery data might benefit more international institutions for QI purposes.

For hospitals that participate in both the ACS-NSQIP and STS-ACSD, use of the STS-ACSD for QI efforts in cardiac surgery is preferred for several reasons: (1) The STS-ACSD obtains data from all cardiac operations at contributing institutions, while the ACS-NSQIP only samples 10–15% of operations; (2) since the ACS-NSQIP deidentifies their national database, there is no way to know the types of institutions contributing cardiac surgery data; and (3) the results of our study show that there are significantly more patient data (670 830 vs. 28 912 operations) in the STS-ACSD, and the STS-ACSD models have superior performance compared with the models computed from the ACS-NSQIP database.

The STS-ACSD preoperative predictor models had higher c-indexes than the ACS-NSQIP models for potentially several reasons. First, the sample sizes for the prediction models were much larger in the STS-ACSD vs. the ACS-NSQIP. The total number of potential predictor variables for inclusion in the backward selection model was significantly higher in the STS-ACSD compared to the ACS-NSQIP (65 covariates vs. 28)^10^. Third, while prior literature has shown preoperative laboratory values are not important and often missing for predicting postoperative outcomes in non-cardiac surgery^16^, this is untested for cardiac operations. The ACS-NSQIP cardiac models may have been improved with the addition of laboratory variables as potential predictors in the backward selection model. And fourth, the candidate predictor variables included in the STS-ACSD models were more disease-specific and operation-specific for patients undergoing cardiac surgery than the ACS-NSQIP predictors. For example, while the ACS-NSQIP PUF includes “congestive heart failure within 30 days^16^, ” a covariate specifically related to cardiac function, there are a considerable number of variables specific to heart function and health in the STS-ACSD models (e.g. ejection fraction, preoperative intra-aortic balloon pump, presence of left main coronary artery disease, endocarditis, cardiac arrythmia and type, heart failure class and timing, among others). While the number and specificity of these cardiac predictor variables make the STS-ACSD risk calculator more burdensome to use, these more specific predictors likely contribute to its models’ superior performance.

Strengths of the study include: (1) to the authors’ knowledge, this was the first attempt to perform a comprehensive analysis of the cardiac operations in the ACS-NSQIP to analyze outcome rates and generate preoperative predictor models for postoperative cardiac surgery outcomes; (2) we analyzed cohorts that were similar to the three cohorts analyzed by the STS, which made for more accurate comparison between the two datasets and their outcomes and predictor models. Limitations of this study include: (1) a small sample size used to develop the ACS-NSQIP models; (2) the ACS-NSQIP data are completely deidentified, so we have no knowledge about which hospitals contributed data to the cardiac dataset; (3) we did not compare risk-adjusted outcomes between the two databases, because we did not have access to the STS-ACSD data; (4) we did not compare the postoperative outcomes of patients who underwent aortic operations, despite the fact that aortic operations make up a significant portion of cardiac surgery operations in the STS-ACSD; this could be an area for future research; (5) patient characteristics from the development cohort in STS-ASCD were not readily available, so it was uncertain if the developmental cohorts were similar in the ACS-NSQIP and STS-ACSD; and (6) lack of cardiac-specific preoperative predictors in the ACS-NSQIP may have resulted in models that were inferior to those generated using the STS-ACSD.

In conclusion, we successfully analyzed the ACS-NSQIP to estimate unadjusted rates of postoperative outcomes and to develop models to predict postoperative outcomes for cardiac surgery and compared these to the STS-ACSD models. Most postoperative outcome rates were similar to those reported by the STS even when statistically different. The ACS-NSQIP models performed almost as well as STS-ACSD preoperative prediction models. The STS-ACSD models possibly performed better due to larger sample size and use of more preoperative variables and more that were disease-specific. However, the ACS-NSQIP models did show good prediction and calibration for a number of outcomes and operation groups. The ACS-NSQIP could be used for study of cardiac surgery outcomes for institutions without access to the STS-ACSD.

## Ethical oversight statement

The Colorado Multiple Institutional Review Board determined this study exempt from review as it used publicly available deidentified data.

## Source of funding

This work was supported by an internal grant from the Department of Surgery, University of Colorado School of Medicine.

## Author contribution

A.R.D., W.G.H., and R.A.M. conceived the study and design. M.R.B. and C.M.S. contributed to data collection. N.P., K.L.C., and J.C.C. contributed to interpretation of the data. A.R.D. wrote the original draft. All authors contributed to critical revisions.

## Conflicts of interest disclosure

The authors report no conflicts of interest. The ACS-NSQIP and participating hospitals are the source of these data; they have not verified and are not responsible for the statistical validity of the data analysis or the conclusions derived by the authors.

## Guarantor

Authors Dyas and Meguid accept full responsibility for the work and conduct of the study, had access to the data, and controlled the decision to publish.

## Provenance and peer review.

Not commissioned, externally peer-reviewed.

## Supplementary Material
