# Supplementary material for: A comparison of the National Surgical Quality Improvement Program and the Society of Thoracic Surgery Cardiac Surgery preoperative risk models: a cohort study
Source: Int J Surg. 2023 May 18;109(8):2334–43. doi: 10.1097/JS9.0000000000000490 (PMC10442082; doi:10.1097/JS9.0000000000000490)
Supplement: Supplementary file 1 [file js9-109-2334-s001.docx]

|  | **Model HL** | **Cross validate HL** |
| --- | --- | --- |
|  | **c-index: 0663 (0.633-0.693) Brier score: 0.0163** | **c-index: 0.648 (0.618-0.678) Brier score: 0.0164** |
| **Stroke** | 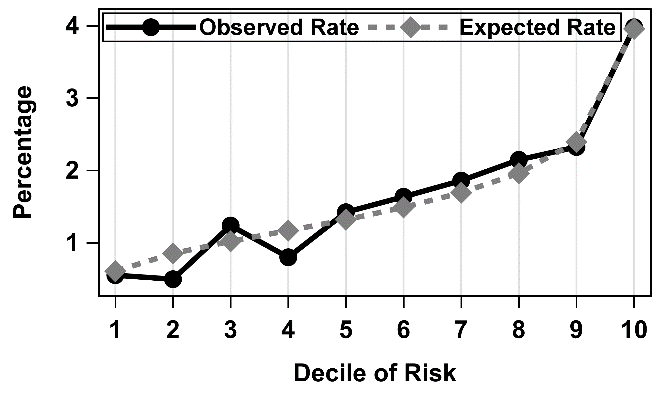 | 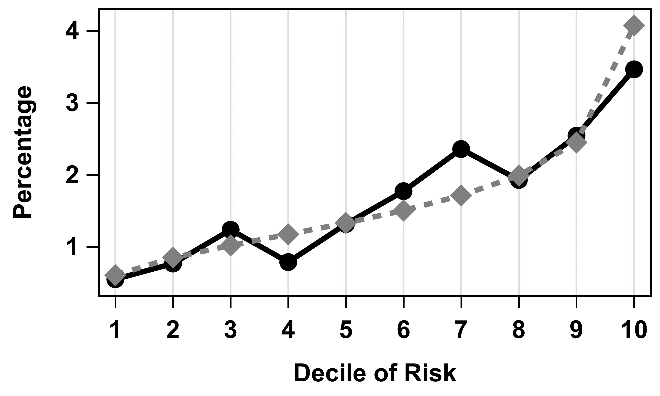 |
|  | **c-index: 0.729 (0.693-0.764) Brier score: 0.0263** | **c-index: 0.713 (0.676-0.749) Brier score: 0.0265** |
| **Renal Failure** | 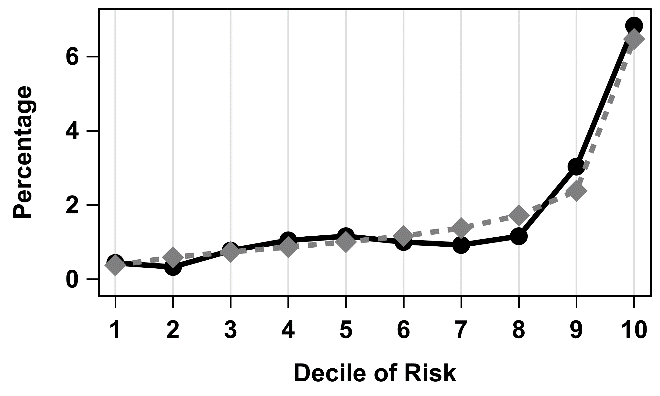 | 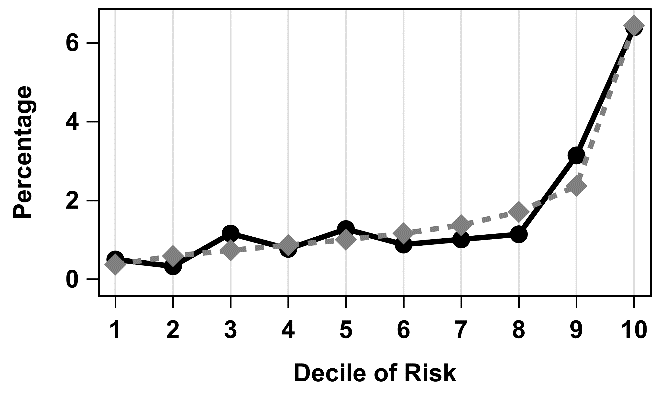 |
|  | **c-index: 0.722 (0.676-0.768) Brier score: 0.0412** | **c-index: 0.695 (0.648-0.7413) Brier score: 0.0423** |
| **Ventilator Dependent** | 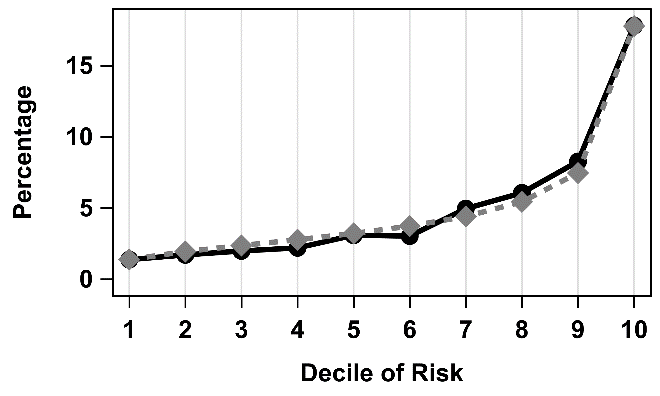 | 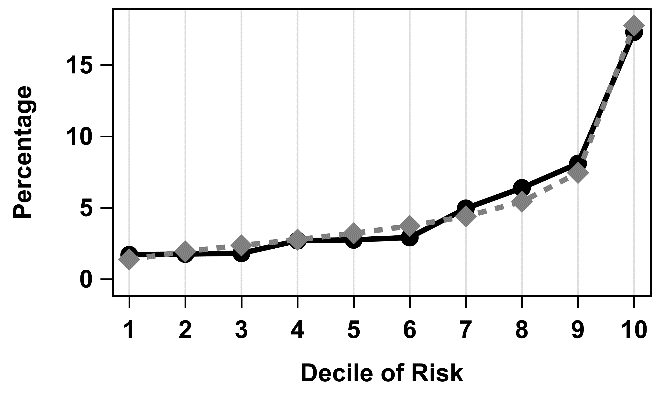 |
|  | **c-index: 0.722 (0.676-0.768) Brier score: 0.0412** | **c-index: 0.695 (0.648-0.7413) Brier score: 0.0423** |
| **Sternal Infection** | 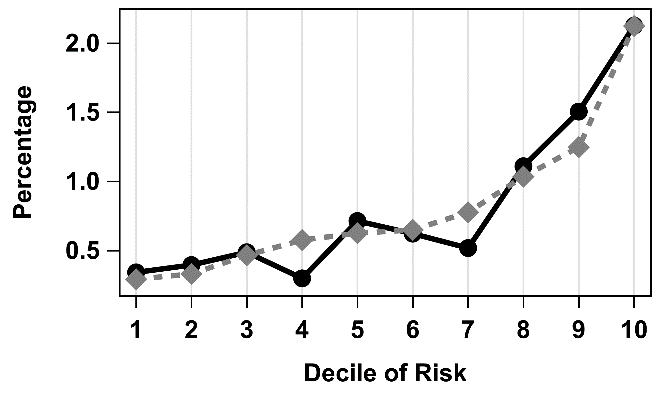 | 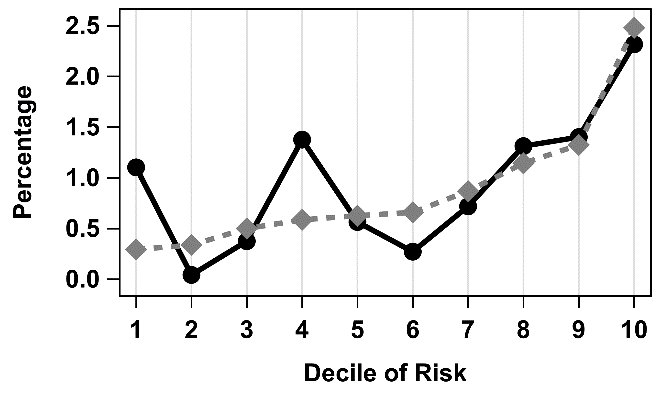 |
|  | **c-index: 0.722 (0.676-0.768) Brier score: 0.0412** | **c-index: 0.695 (0.648-0.7413) Brier score: 0.0423** |
| **Return to OR** | 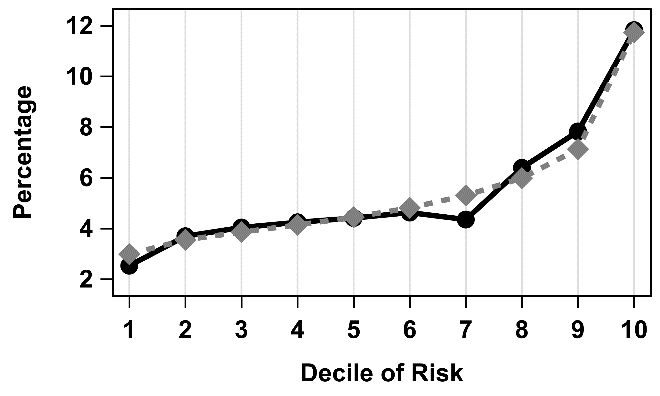 | 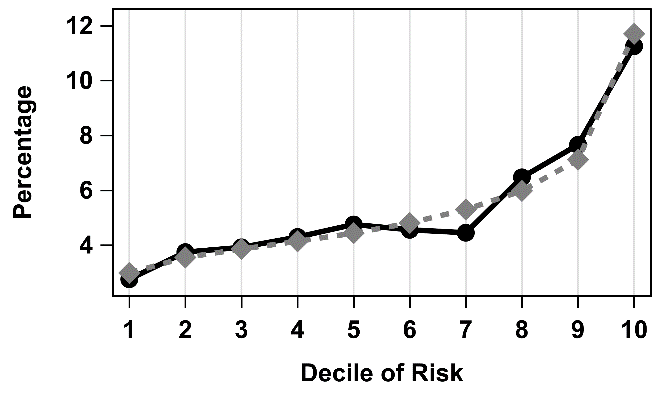 |
|  | **c-index: 0.722 (0.676-0.768) Brier score: 0.0412** | **c-index: 0.695 (0.648-0.7413) Brier score: 0.0423** |
| **Composite M&M** | 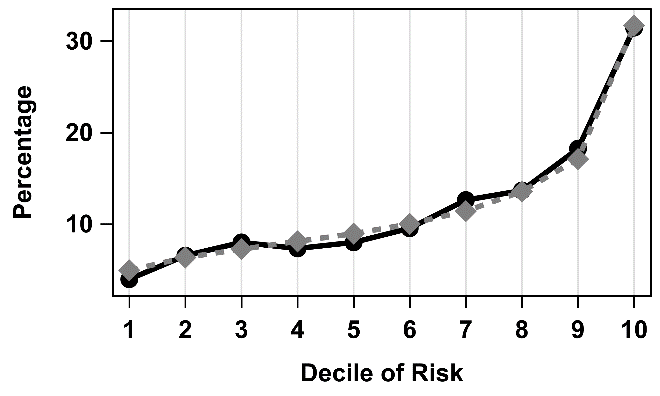 | 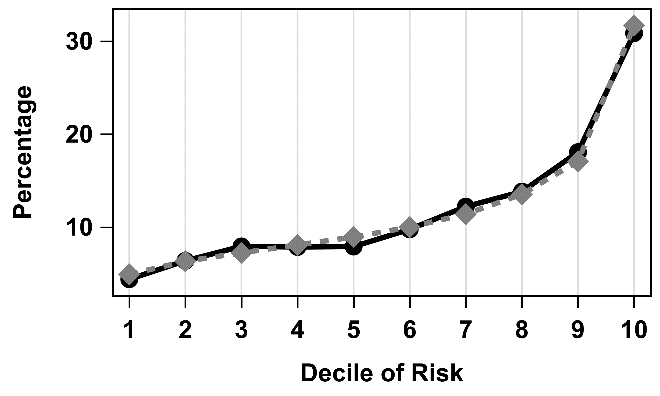 |
|  | **c-index: 0.722 (0.676-0.768) Brier score: 0.0412** | **c-index: 0.695 (0.648-0.7413) Brier score: 0.0423** |
| **LOS > 14 days** | 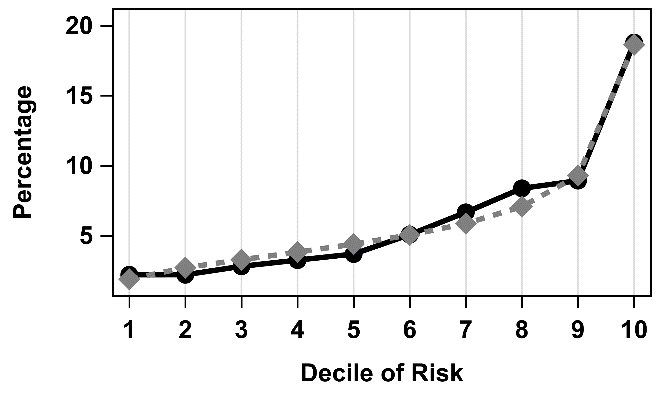 | 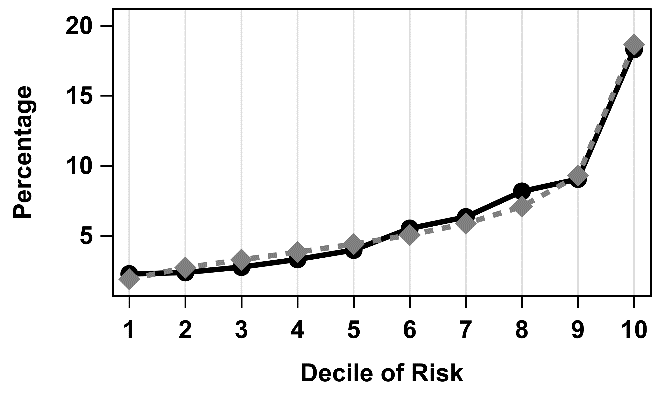 |
|  | **c-index: 0.722 (0.676-0.768) Brier score: 0.0412** | **c-index: 0.695 (0.648-0.7413) Brier score: 0.0423** |
| **LOS < 6 days** | 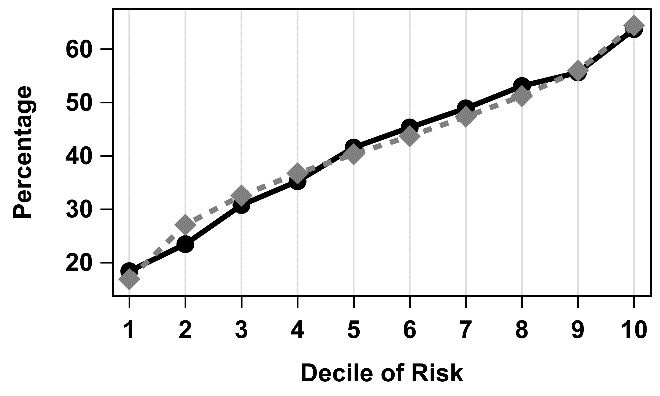 | 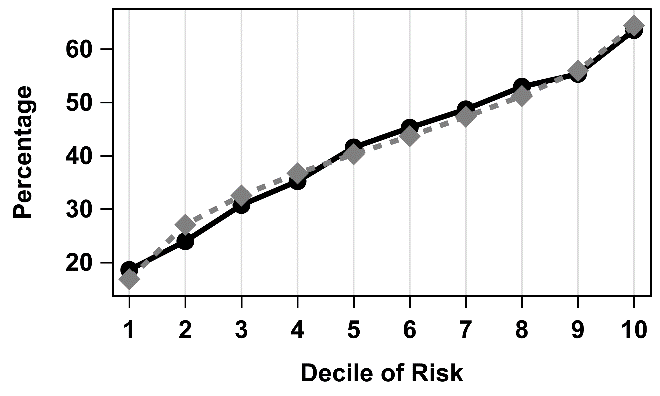 |
| **Abbreviation: HL, Hosmer-Lemeshow: OR, Operating Room; LOS, Length of Stay; M&M, morbidity and mortality** | | |
