# Supplementary material for: A comparison of the National Surgical Quality Improvement Program and the Society of Thoracic Surgery Cardiac Surgery preoperative risk models: a cohort study
Source: Int J Surg. 2023 May 18;109(8):2334–43. doi: 10.1097/JS9.0000000000000490 (PMC10442082; doi:10.1097/JS9.0000000000000490)
Supplement: Supplementary file 3 [file js9-109-2334-s003.docx]

Supplemental Figure 2. Hosmer-Lemeshow Partitions Valve Only.

|  | **Model HL** | **Cross validate HL** |
| --- | --- | --- |
|  | **c-index: 0.682 (0.636-0.729) Brier score: 0.0164** | **c-index: 0.655 (0.607-0.704) Brier score: 0.0164** |
| **Stroke** | 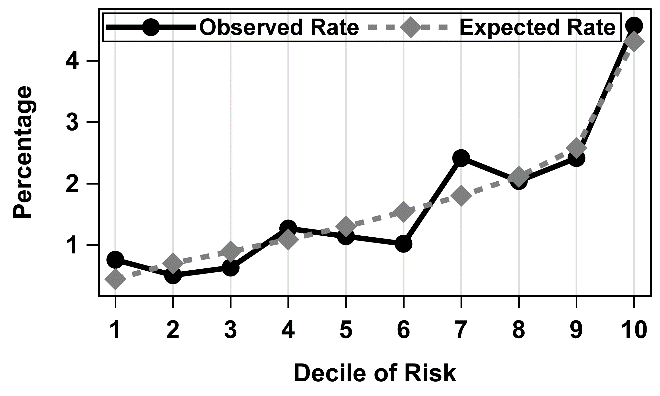 | 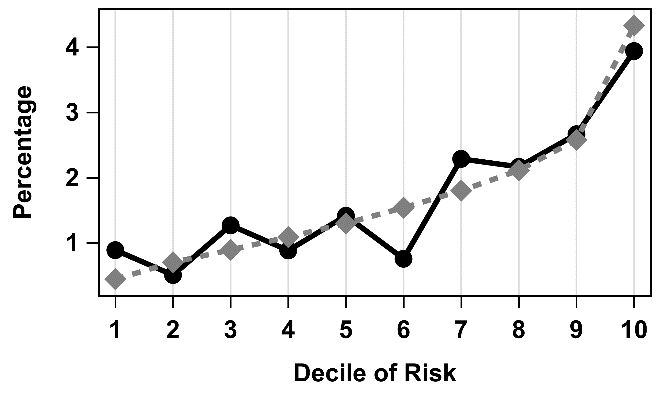 |
|  | **c-index: 0.765 (0.727-0.804) Brier score: 0.0215** | **c-index: 0.753 (0.714-0.792) Brier score: 0.0218** |
| **Renal Failure** | 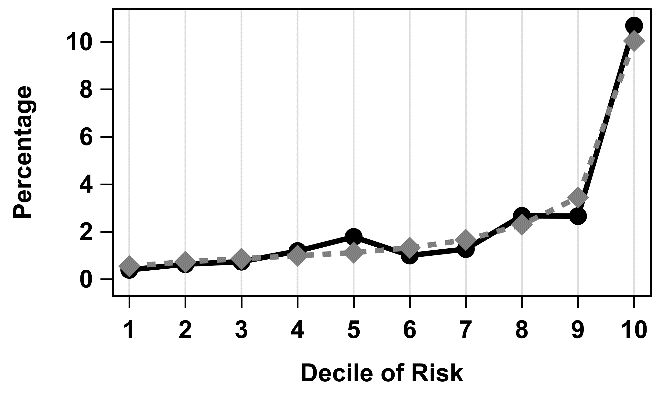 | 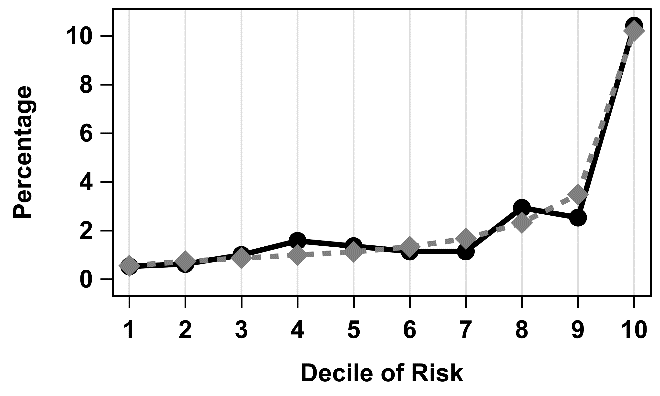 |
|  | **c-index: 0.749 (0.727-0.772) Brier score: 0.0556** | **c-index: 0.742 (0.719-0.764) Brier score: 0.0562** |
| **Ventilator Dependent** | 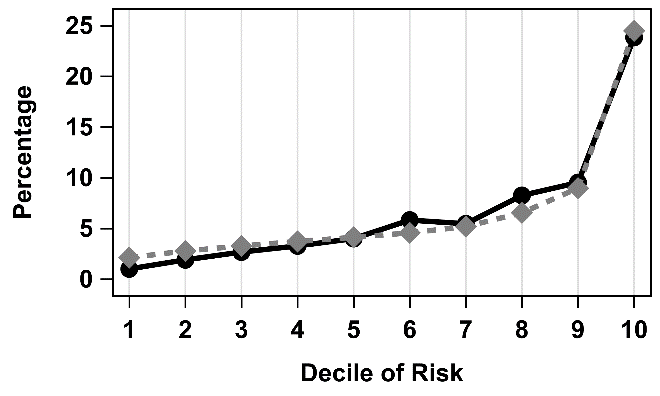 | 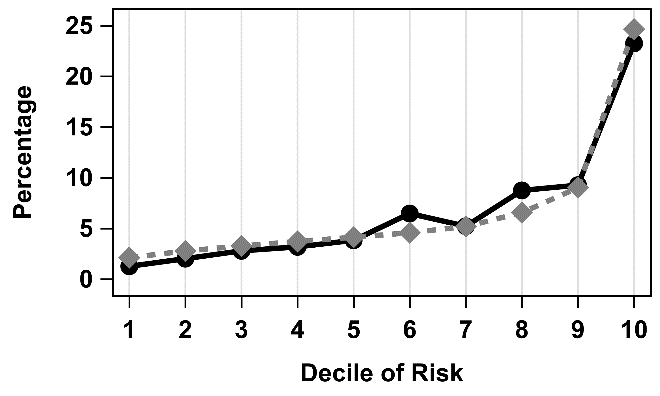 |
|  | **c-index: 0.709 (0.639-0.778) Brier score: 0.0053** | **c-index: 0.572 (0.489-0.655) Brier score: 0.0053** |
| **Sternal Infection** | 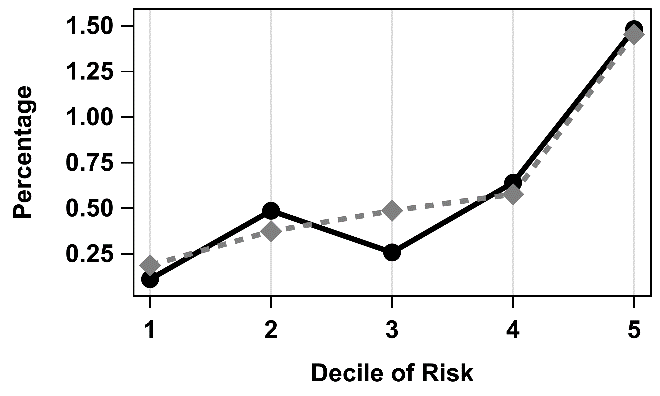 | 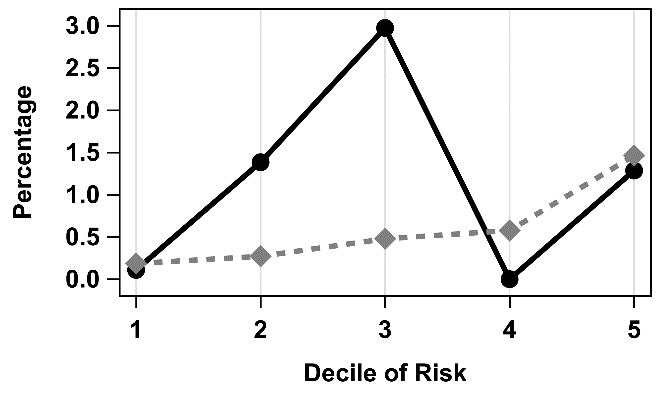 |
|  | **c-index: 0.605 (0.582-0.628) Brier score: 0.0731** | **c-index: 0.592 (0.569-0.615) Brier score: 0.0735** |
| **Return to OR** | 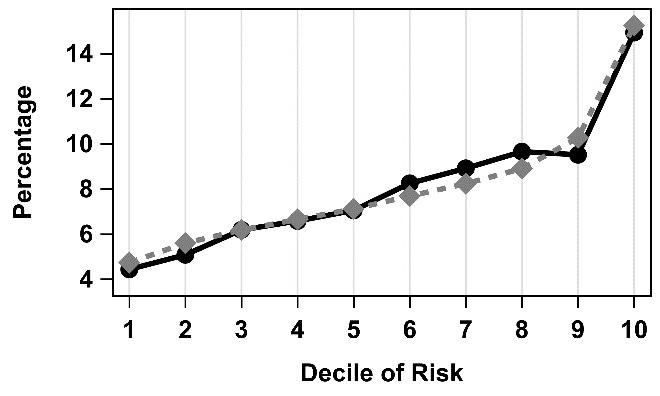 | 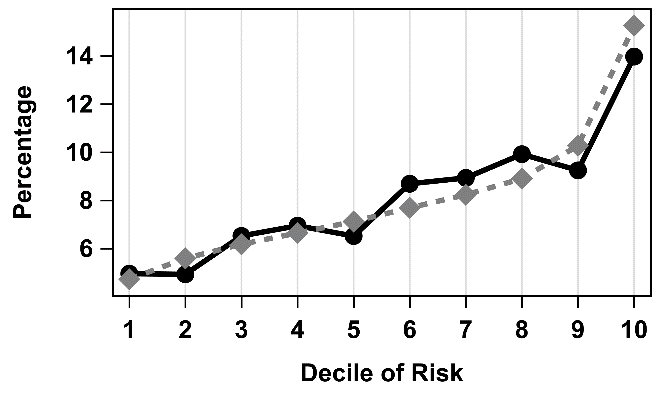 |
|  | **c-index: 0.683 (0.667-0.700) Brier score: 0.1221** | **c-index: 0.677 (0.660-0.694) Brier score: 0.1232** |
| **Composite M&M** | 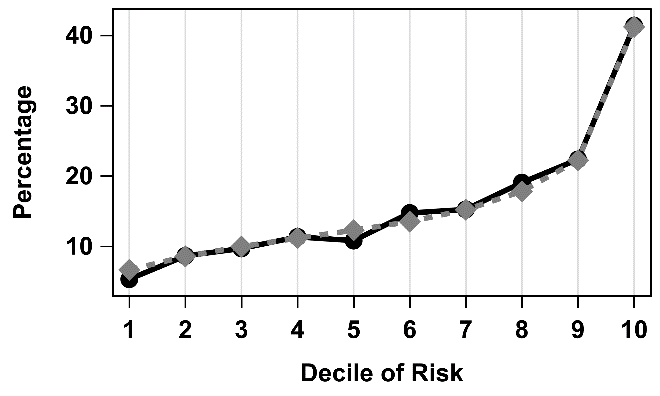 | 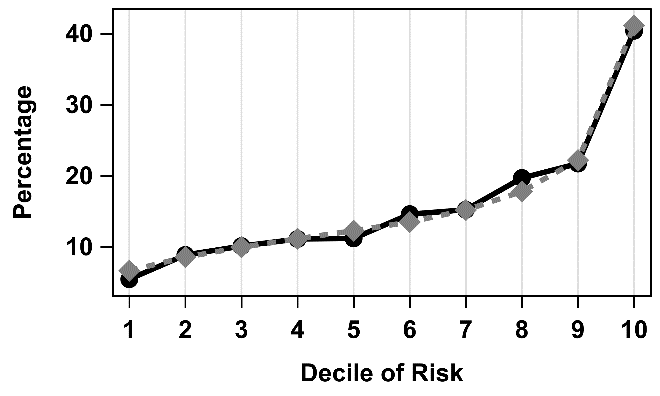 |
|  | **c-index: 0.737 (0.718-0.756) Brier score: 0.0783** | **c-index: 0.728 (0.709-0.748) Brier score: 0.0793** |
| **LOS > 14 days** | 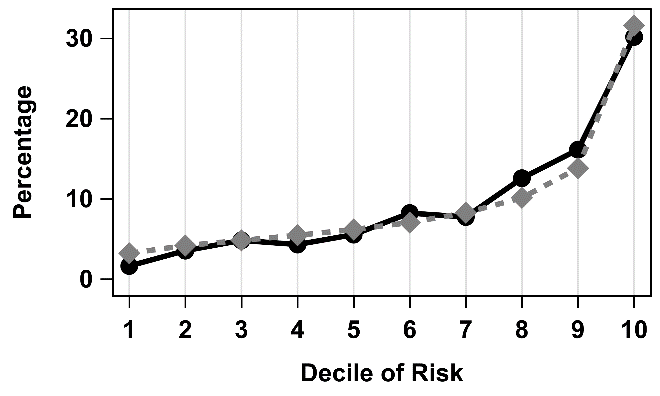 | 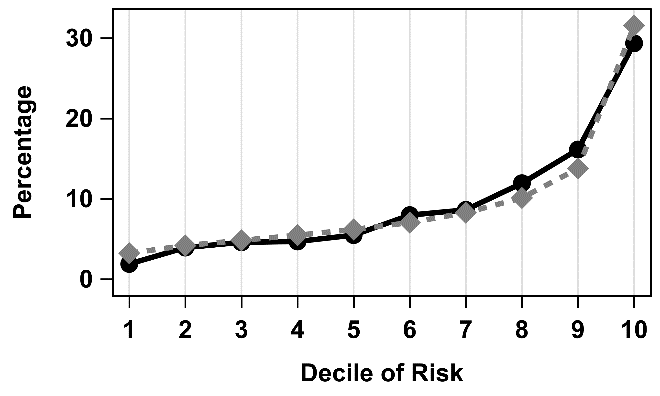 |
|  | **c-index: 0.675 (0.662-0.687) Brier score: 0.2058** | **c-index: 0.670 (0.657-0.682) Brier score: 0.2069** |
| **LOS < 6 days** | 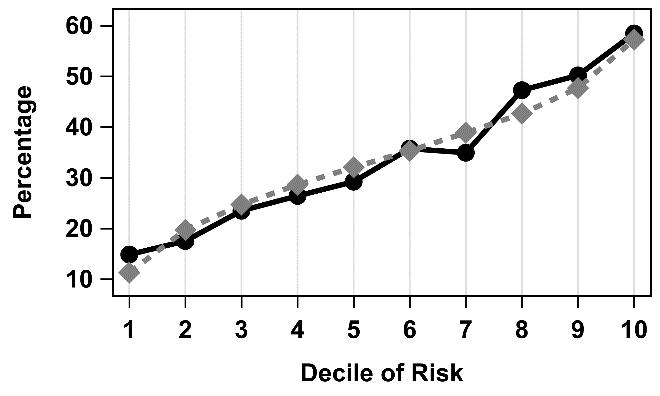 | 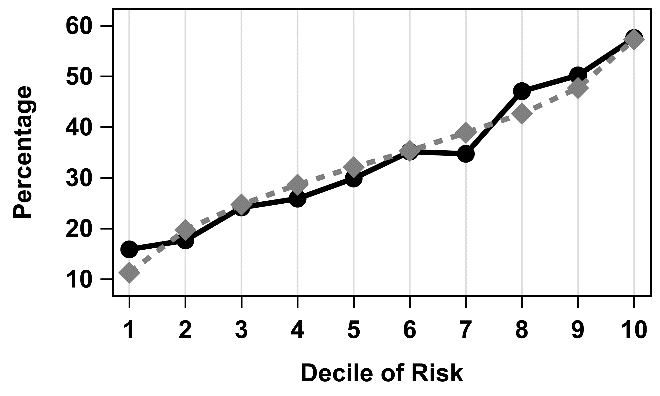 |
| **Abbreviation: HL, Hosmer-Lemeshow: OR, Operating Room; LOS, Length of Stay; M&M, morbidity and mortality** | | |
