# Supplementary material for: A comparison of the National Surgical Quality Improvement Program and the Society of Thoracic Surgery Cardiac Surgery preoperative risk models: a cohort study
Source: Int J Surg. 2023 May 18;109(8):2334–43. doi: 10.1097/JS9.0000000000000490 (PMC10442082; doi:10.1097/JS9.0000000000000490)
Supplement: Supplementary file 4 [file js9-109-2334-s004.docx]

|  | **Model HL** | **Cross validate HL** |
| --- | --- | --- |
|  | **c-index: 0.593 (0.531-0.654) Brier score: 0.0289** | **c-index: 0.571 (0.510-0.633) Brier score: 0.0291** |
| **Stroke** | 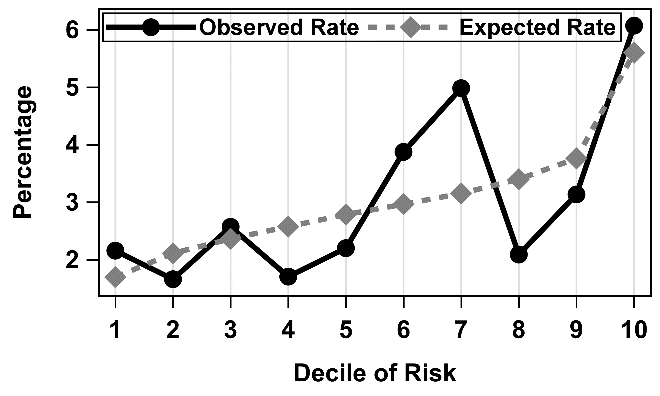 | 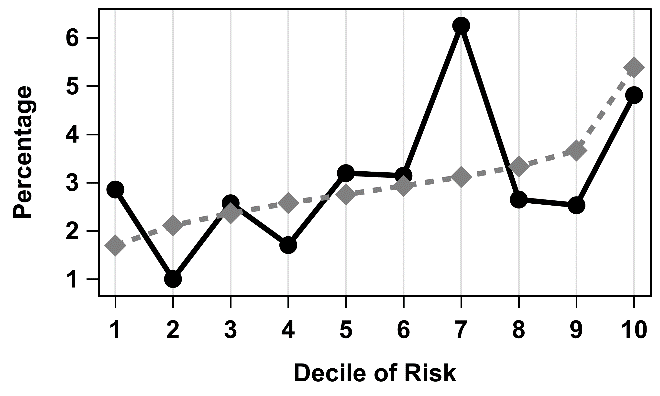 |
|  | **c-index: 0.732 (0.684-0.779) Brier score: 0.0350** | **c-index: 0.711 (0.662-0.759) Brier score: 0.0358** |
| **Renal Failure** | 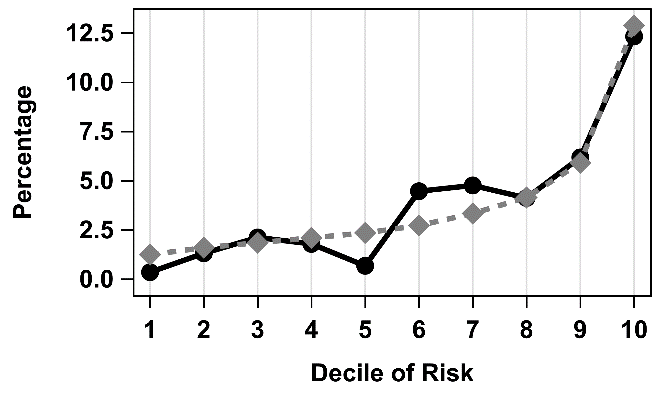 | 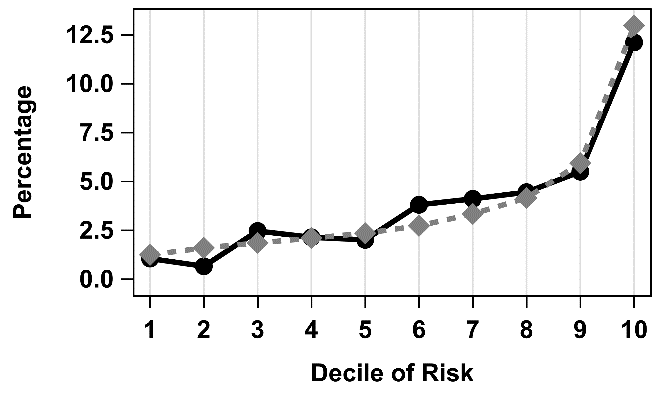 |
|  | **c-index: 0.735 (0.706-0.763) Brier score: 0.0913** | **c-index: 0.714 (0.685-0.743) Brier score: 0.0939** |
| **Ventilator Dependent** | 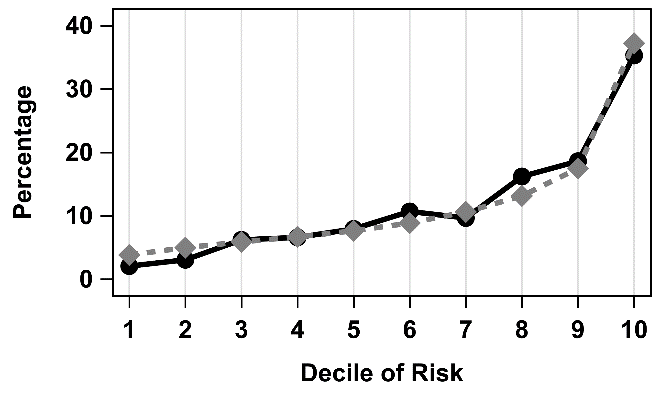 | 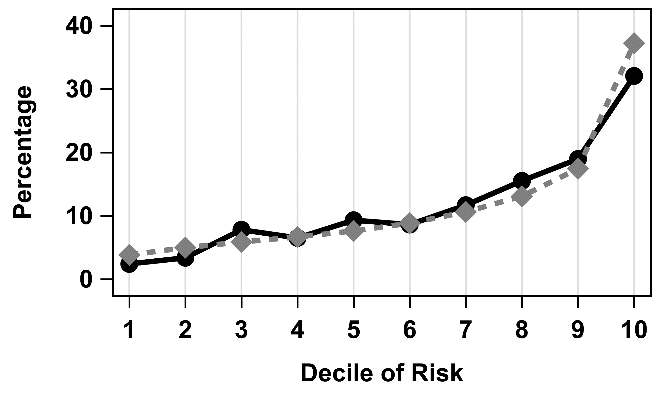 |
|  | **c-index: 0.775 (0.689-0.861) Brier score: 0.0076** | **c-index: 0.664 (0.547-0.780) Brier score: 0.0078** |
| **Sternal Infection** | 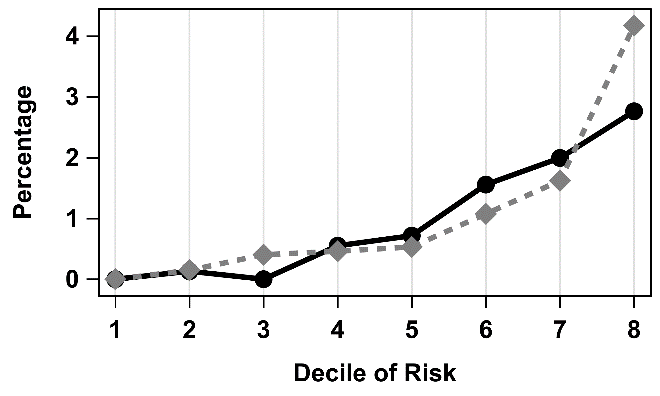 | 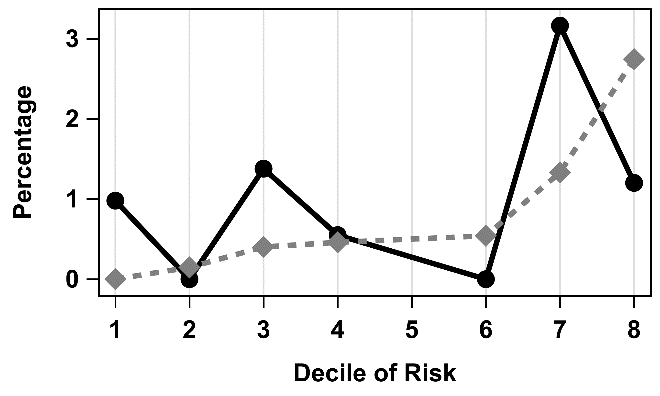 |
|  | **c-index: 0.582 (0.547-0.616) Brier score: 0.0926** | **c-index: 0.567 (0.533-0.602) Brier score: 0.0930** |
| **Return to OR** | 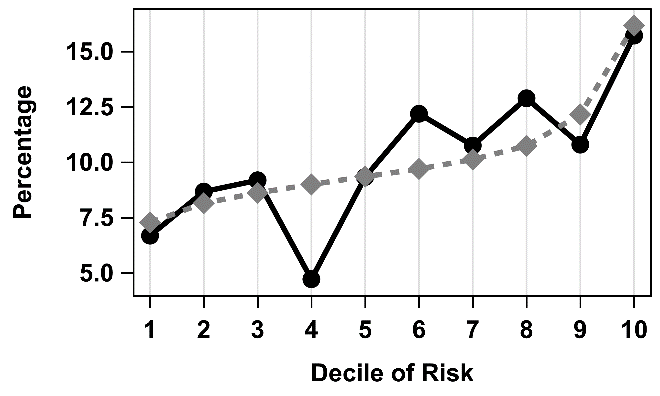 | 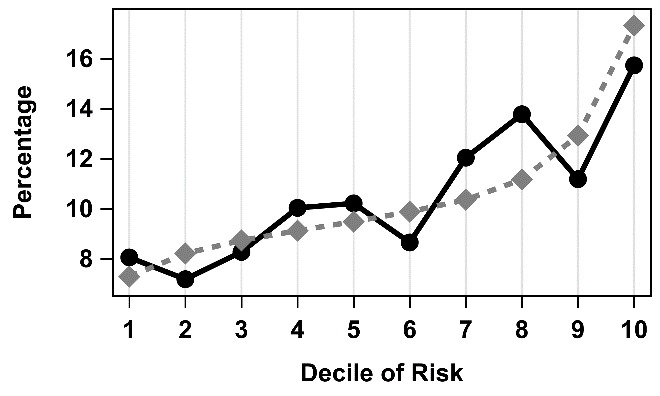 |
|  | **c-index: 0.661 (0.637-0.686) Brier score: 0.1627** | **c-index: 0.650 (0.625-0.674) Brier score: 0.1650** |
| **Composite M&M** | 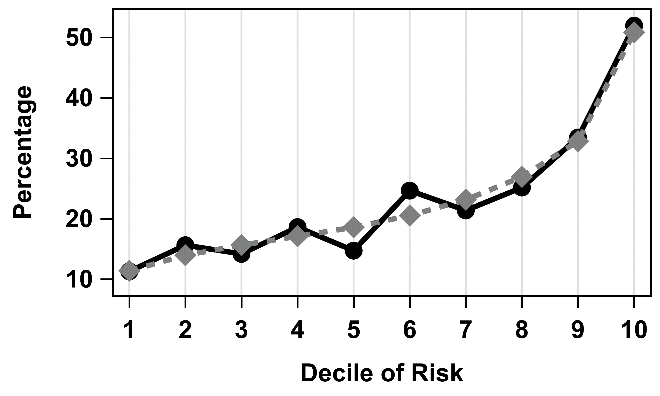 | 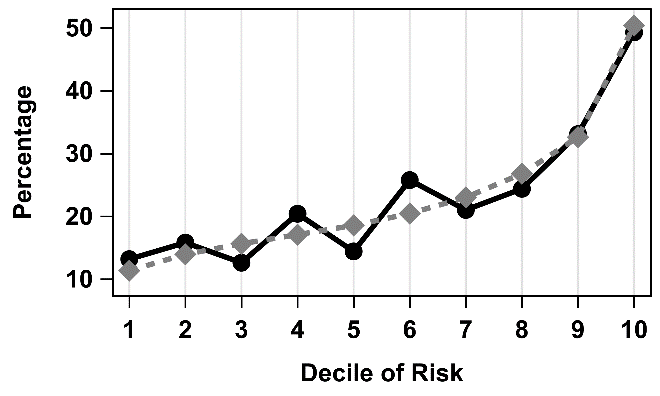 |
|  | **c-index: 0.691 (0.663-0.718) Brier score: 0.1194** | **c-index: 0.677 (0.650-0.04 Brier score: 0.1212** |
| **LOS > 14 days** | 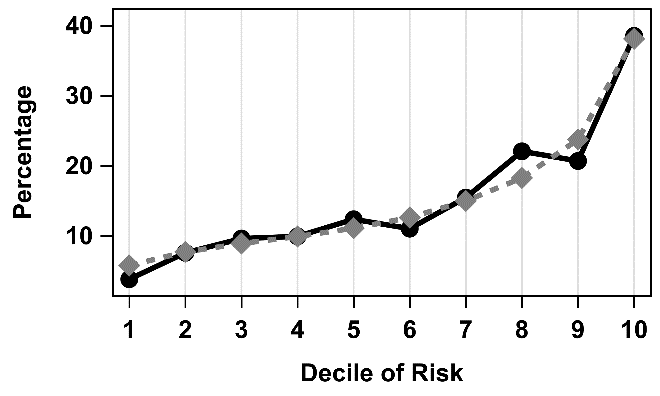 | 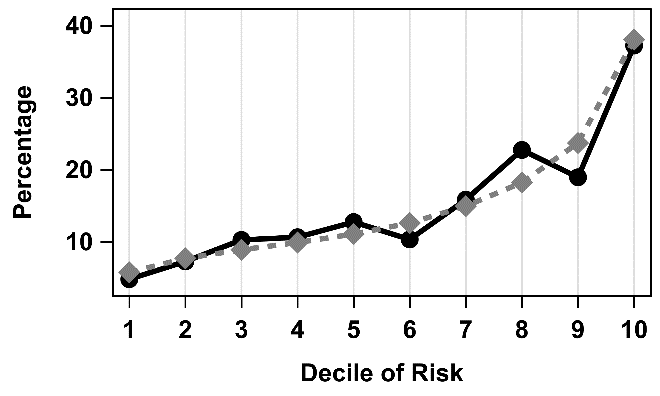 |
|  | **c-index: 0.649 (0.624-0.673) Brier score: 0.1603** | **c-index: 0.637 (0.613-0.662) Brier score: 0.1615** |
| **LOS < 6 days** | 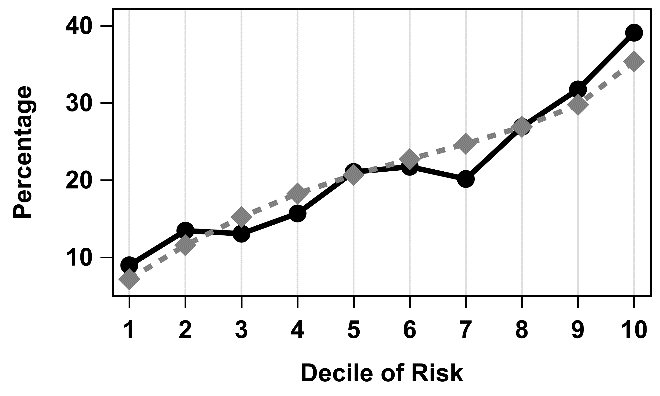 | 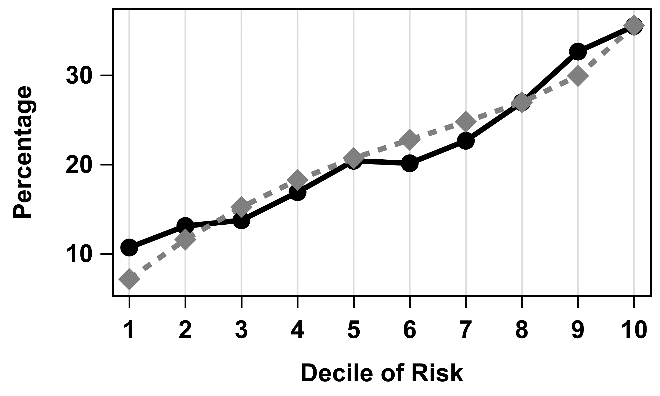 |
| **Abbreviation: HL, Hosmer-Lemeshow: OR, Operating Room; LOS, Length of Stay; M&M, morbidity and mortality** | | |
